# Do Seaweeds Contribute to Nutritional Composition and Acceptance in Traditional Portuguese Recipes?

**DOI:** 10.3390/foods14111947

**Published:** 2025-05-29

**Authors:** Maria Lassalete Mendes, António Pires, Amparo Gonçalves, Carla Pires, Helena Maria Lourenço, Ariana Saraiva, Renata Puppin Zandonadi, Fernando Ramos, António Raposo

**Affiliations:** 1 Estoril School of Hospitality and Tourism, Av. Condes de Barcelona 808, 2769-510 Estoril, Portugal; maria.mendes@eshte.pt (M.L.M.); antonio.pires@eshte.pt (A.P.); 2 Division of Aquaculture, Valorization and Bioprospecting, Department for the Sea and Marine Resources, Portuguese Institute for the Sea and Atmosphere (IPMA, I.P.), Av. Dr. Alfredo Magalhães Ramalho, 6, 1495-165 Lisboa, Portugal; amparo@ipma.pt (A.G.); cpires@ipma.pt (C.P.); helena@ipma.pt (H.M.L.); 3 Interdisciplinary Center of Marine and Environmental Research (CIIMAR), University of Porto, Terminal de Cruzeiros de Leixões, Av. General Norton de Matos s/n, 4450-208 Matosinhos, Portugal; 4 Research in Veterinary Medicine (I-MVET), Faculty of Veterinary Medicine, Lisbon University Centre, Lusófona University, Campo Grande 376, 1749-024 Lisboa, Portugal; ariana.saraiva@ulusofona.pt; 5 Veterinary and Animal Research Centre (CECAV), Faculty of Veterinary Medicine, Lisbon University Centre, Lusófona University, Campo Grande 376, 1749-024 Lisboa, Portugal; 6 Nutrition Department, Faculty of Health Sciences, University of Brasília, Campus Universitário Darcy Ribeiro, Brasilia 70910-900, Brazil; renatapz@unb.br; 7 Faculty of Pharmacy, University of Coimbra, Azinhaga de Santa Comba, 3000-548 Coimbra, Portugal; framos@ff.uc.pt; 8 Associated Laboratory for Green Chemistry (LAQV) of the Network of Chemistry and Technology (REQUIMTE), Rua D. Manuel II, Apartado 55142, 4051-401 Porto, Portugal; 9 CBIOS (Research Center for Biosciences and Health Technologies), Universidade Lusófona de Humanidades e Tecnologias, Campo Grande 376, 1749-024 Lisboa, Portugal

**Keywords:** acceptance, edible seaweeds, potassium, sodium, sustainability

## Abstract

Consumers’ growing concern about sustainability and health affects their food choices as long as there is acceptance in terms of sensory aspects. The challenge of finding new sustainable food sources with a smaller ecological footprint makes seaweed a candidate for human consumption, considering that they are poorly exploited marine food resources in European countries. Therefore, this study aimed to evaluate the effect of the inclusion of different seaweeds (wakame and sea spaghetti) in three traditional Portuguese recipes, namely octopus salad (SP and SPW), monkfish rice with prawns (AT and ATW) and stewed cuttlefish with white beans and clams (FC and FCE), regarding their acceptance and nutritional aspects. Sensory and physicochemical analyses were carried out using reference methods. The results showed that the modified recipes with seaweeds (SPW, ATW, and FCE) were well accepted by a nontrained sensory panel and did not change nutritional aspects in terms of macronutrient content, ash, and sodium. However, the inclusion of wakame contributed to an increase in the potassium content in octopus salad (SPW) and monkfish rice (ATW). In short, sensory results highlighted the potential for seaweed inclusion in Portuguese traditional recipes without compromising its identity. Future work should evaluate the partial substitution of fish/mollusks with seaweed in traditional recipes to improve the sustainability and nutritional contribution of these recipes.

## 1. Introduction

Globalization has raised questions regarding the need for each country to be self-sufficient and feed its population by producing new protein sources with controlled costs. Since oceans have the largest representation on the planet, they are expected to be an integral part of the response. In some European countries, the challenge of finding new sustainable food sources with a smaller ecological footprint than those used today makes seaweed a candidate for human consumption, considering that they are a generous and poorly exploited marine food resource [1,2,3,4,5,6].

Seaweeds are photosynthetic plant-like eukaryotic organisms from oceans that are classified into three main groups (red, known as Rhodophyta; brown, known as Phaeophyceae; and green, known as Chlorophyta). Seaweeds are considered one of the planet’s most significant renewable global biomass resources and have the potential to contribute toward a sufficient and healthy global food supply for a population expected to grow exponentially [3,6,7]. In this sense, several projects have emerged to evaluate the benefits of using seaweed in human nutrition, searching for natural nutrient sources and bioactive compounds to help populations’ health. Seaweeds represent one of the largest sources of bioactive components found in abundance in oceanic environments [1,8,9,10,11].

Although seaweeds’ chemical composition varies across species, harvesting seasons, and eco-habitat, the content of nutrients and bioactive compounds in these organisms, including pigments, fatty acids, sterols, proteins, lipids, fibers, polysaccharides, fucoxanthin, vitamins, and minerals, in addition to their pleasant umami flavor, highlights seaweeds’ application in food products with a potential nutritional contribution [4,5,6,12]. Some species have a high protein amount (113–123 g/kg db) (in general, red species tend to present high levels of proteins (with all essential amino acids), green species have moderate levels, and brown species present low protein levels [13]). They also contribute to the daily intake of vitamin C [4,6,14], are low in fat and calories but rich in minerals such as iron and calcium, and present selenium and polysaccharides [3,4,11,12,13,14,15,16]. In this sense, adding seaweed to any regularly consumed food, if properly processed, can improve consumers’ health due to the amount of macro- and micronutrients available. However, to exert positive human health effects, their components must be digestible and absorbed to achieve the target levels, driven by their bioavailability [13,17]. For example, a review study reported that some animal proteins, such as milk and eggs, present the highest digestibility values (about 95%), and the digestibility of seaweed protein varies from 5 to 86% [13,16,18,19]. Some seaweeds present reduced protein digestion mainly because of their high soluble fiber content, which can impair access to proteolytic enzymes [13]. However, heating, pressure, fermentation, enzymatic treatments, or electro-based technologies may improve protein digestibility and bioavailability, improving the properties of seaweed protein [13,17]. In addition, studies show that when seaweeds are used in recipes, they can be a healthy alternative for reducing salt use, a problem that has affected the populations of industrialized countries [19,20,21,22].

Therefore, interest in these “sea vegetables” has grown exponentially in recent years, mainly among scientists, chefs, and companies dedicated to understanding and commercializing these products [23,24]. The bioactive components found in seaweeds, in addition to being beneficial to health, can be used in the food industry as ingredients, thus opening up new fields of action [25]. Also, there may be a reduction in the use of chemical preservatives when macroalgae and their extracts are applied in the food industry [15,26]. They have been used in the industrial sector as food, fuel, plastics, and cosmetics and in the pharmaceutical industry due to their antioxidant, anti-inflammatory, antimutagenic, antitumor, antidiabetic, and antihypertensive properties, through specific cellular and molecular mechanisms [27].

Although its potential use has been increasing in the West in recent years, interest in seaweed as a food source is only a tiny fraction compared to the East, particularly in Asian countries such as Japan, where a fifth of meals contain seaweed. Although Asian products currently dominate the European seaweed market, opportunities for more local products are arising, guided by novelty and growing consumer demand for local and traceable food products [6,28]. Concerning strategies to use seaweeds in culinary applications, including them in existing products may result in better acceptance, thus favoring seaweed inclusion to achieve the desired results in a shorter time, as shown previously with its addition to items such as snacks, bread, and soups [3,5,29]. Environmental sustainability, health, and gastronomic versatility are some of the potential benefits of consuming seaweeds [3,29]. Regions of the European coast, such as Galicia, French Brittany, Wales, and Ireland, are rich in edible seaweeds, which are distinguished by the variety and quantity of species. Galicia can be used as a reference for Portugal for three relevant reasons: geographic proximity, the numerous studies carried out, and the existence of methods for the extraction and processing of seaweeds for use in food products [1,30].

Since Portugal has the privilege of having a unique coastline characterized by an immense variety of marine fauna and flora, edible seaweeds have raised interest in the Portuguese food industry, resulting in the need for greater knowledge of their characteristics and physical–chemical properties that can add nutritional and economic value to Portuguese gastronomy [1,23,30]. In Portugal, the application of seaweeds can play an important role in sustainability and health, but it is still challenging to include them in the population’s diet by innovating traditional recipes without altering their identity and acceptance [1,23,30].

Given consumers’ growing concern about sustainability and health, they influence their food choices as long as there is acceptance regarding sensory aspects. In this sense, this study hypothesized that seaweeds contribute to nutritional composition in traditional Portuguese recipes without reducing their acceptance. Therefore, this study aimed to evaluate the effect of seaweeds’ inclusion (wakame and sea spaghetti) in three traditional Portuguese recipes, namely octopus salad (SP and SPW), monkfish rice with prawns (AT and ATW), and stewed cuttlefish with white beans and clams (FC and FCE), regarding their acceptance and nutritional aspects.

## 2. Materials and Methods

### 2.1. Characteristics of Recipes and Ingredients

The subject of this research was traditional Portuguese recipes included in regional and traditional Portuguese cuisine books, with high acceptance by the Portuguese population, which is the case with octopus salad (*Salada de Polvo, SP*), monkfish rice with prawns (*Arroz de Tamboril com Camarão, AT*), and stewed cuttlefish with white beans and clams (*Feijoada de Choco com Amêijoas, FE*).

The octopus salad (SP) is a traditional Portuguese dish in coastal areas and is prepared with cooked (boiled) octopus cut into slices, chopped onion, green or red pepper (or a mixture of both), fresh coriander or parsley, and seasoned with olive oil and vinegar. It can be used as a starter, snack, or main dish. When the octopus is boiled for the salad, no salt is added [31], as the sodium levels tend to be high due to the nature of the ingredients.

The monkfish rice with prawns (AT) is cooked in a seafood broth previously prepared as follows: prawn heads are sautéed in olive oil, with brandy added to refresh; then, chopped onion, celery, leek, carrots, and garlic are added, braising the mixture for a while. Then, tomato pulp, monkfish spines, and water are added and left to cook for 20 min. Monkfish muscle cut into cubes is cooked in the seafood broth, with aromatic herbs such as coriander, white wine, salt, and pepper. The Portuguese Carolino variety of rice and raw prawn tails (cut into cubes) are added to the broth and left to cook over low temperature, ensuring adequate rice cooking and firmness of prawn cubes (should be firm).

The stewed cuttlefish with white beans and clams (FC) is generally prepared with cuttlefish cut into cubes, which are stewed in olive oil; onion and garlic are also used, as well as carrots, fresh tomato, salt and pepper, and dry white beans (previously soaked and boiled). The water used to boil the beans is added. During stewing, white wine is used to refresh the preparation, and clam meat is added and left to cook for approximately 5 min.

The variants of those recipes with seaweed inclusion were prepared and evaluated in the Estoril School of Hospitality and Tourism (ESHTE). The ingredients of both variants of each recipe are presented in Table 1. Sea spaghetti (*Himanthalia elongata*) and wakame (*Undaria pinnatifida*) were purchased as dehydrated seaweeds, and they were rehydrated as follows: for each recipe, an amount of 20 g (for approximately 4 persons) was added to a 200 mL tap water (1 g seaweed:10 100 mL water); seaweeds were soaked for 15 min at room temperature (approximately 20 °C). The final weight of rehydrated seaweeds was 100 and 150 g, respectively, for sea spaghetti and wakame, which correspond to a recovery of 5 to 7.5 times the initial weight.

### 2.2. Sensory Analysis

#### 2.2.1. Quantitative Descriptive Method—Sensory Profile

In the first stage, the recipes were subjected to a quantitative descriptive analysis to characterize the sensory properties related to the inclusion of seaweeds at the Laboratory of Sensory Analysis of IPMA, equipped with a test room with 5 individual booths, according to ISO 8589 [32]. The sensory panel comprised 8 panelists aged between 35 and 65 years old (60% women), selected among the IPMA sensory panel trained in quantitative descriptive sensory analysis of fish/seafood products, including edible macroalgae, according to the reference outlined criteria and procedures [20]. The specific training of selected panelists on macroalgae was carried out using fresh and rehydrated red and brown macroalgae. The participation of panelists was voluntary based on informed consent.

Both variants of each recipe were presented in white-coded dishes. Portions of 60 g of each recipe were used per panelist, except in the case of SP and SPW, for which portions of 30g/panelist were used. The recipes were presented in white-coded dishes, and the two variants of each recipe were presented sequentially. Water was used to rinse the mouth between recipes’ tasting.

The panelists assessed different attributes and parameters using a 9-point intensity scale [33], ranging from 0 (absent) to 8 (extremely intense): taste (characteristic of the recipe; marine; salty; sweet; bitter; other such as shellfish, bivalves, fish, acid, spicy, and metallic); seaweed texture (adhesiveness and chewiness); mouth sensations (astringency, other sensations); aftertaste (acid, bitter, sweet, metallic, and other); odor (characteristic of the recipe, sea/marine, and other odors); and perception of the seaweed in the recipe.

#### 2.2.2. Affective Method—Acceptance Test (Overall Liking)

An affective test was carried out voluntarily and with informed consent by untrained panelists at ESHTE. Students, professors, and other school professionals were invited to participate and were informed that the recipes would include fish, shellfish, and seaweed. In total, 52 people attended and filled out the questionnaire to determine the panel, excluding those with potential allergies. Ultimately, 4 people could not be accepted due to shellfish allergy; thus, 48 people were selected for inclusion in the sensory panel with the following profile: 54.7% women; 72.7% students, 16.4% administrative staff, and 9.1% professors. Overall, 69.1% of the participants were in the range of 18–25 years, 18.2% in 36–50 years, 7.3% above 50 years old, and 5.4% in the range of 26–35 years; 72.2% of participants recognized the use of seaweed in gastronomy, and 71.1% had already tasted recipes containing seaweeds, such as miso soup, sushi (with nori), ramen, and risottos.

The overall liking of the modified recipes SPW, ATW, and FCE were assessed. Portions of 30 g (SPW) and 60 g (ATW and FCE) were sequentially presented to the panelists in white-coded dishes. Panelists tasted each recipe and expressed their degree of liking using a 9-point hedonic scale (1—dislike extremely; 5—neither like nor dislike; 9—like extremely) [33]. The panelists were also requested to indicate what they most liked and the most negative attributes of each recipe. Recipes were considered to have good acceptance when the percentage of liking was >70% [34].

### 2.3. Physicochemical Analysis

#### 2.3.1. Sample Constitution

Three independent portions of 100 g (replicates) from each traditional and modified recipe were collected. Each portion was adequately homogenized until a homogeneous mass was obtained. All replicates were stored at −20 °C until analysis.

#### 2.3.2. Proximate Composition

Proximates were determined according to the reference methods described in AOAC [35]. The free fat content was determined by Soxhlet extraction using diethyl ether as a solvent and by weighing the fat residue after drying in an air oven (105 ± 1 °C ULE 500, Memmert, Büchenbach, Germany). The moisture content was determined by drying the sample at 105 ± 1 °C in an oven (ULE 500, Memmert, Büchenbach, Germany) until a constant weight was obtained. The ash content was determined after the incineration of the dried sample (105 ± 1 °C, ULE 500, Memmert, Büchenbach, Germany) in a muffle furnace at 500 ± 25 °C (MR 170 E, Heraeus, Hanau, Germany) until a constant weight was obtained. The protein content was determined as total nitrogen using Leco model FP-528 equipment (LECO Corp., St. Joseph, MI, USA), which is released by combustion, at 850 °C, in the presence of oxygen, resulting in nitrogen oxide. Nitrogen was detected by thermal conductivity and was converted into protein using the factor of 6.25.

Total carbohydrates were calculated by difference, according to Equation (1) [36]:

(1) % Total carbohydrates=100−(% moisture+% fat+% ashes+% protein)

Energy value was calculated according to the FAO [36], as expressed in Equation (2):

(2) $$Energy\;value(kcal/100g)=\left(\%\;Carbohydrates\times 4\right)+\left(\%\;Lipids\times 9\right)+(\%\;protein\times 4)$$

#### 2.3.3. Macroelements

The content of Na and K elements was determined by flame atomic absorption spectrometry after dry ashing, based on the method described in Jorhem [37]. The sample was dried in an air oven (ULE 500, Memmert, Büchenbach, Germany) and incinerated in a muffle furnace (MR 170 E, Heraeus, Hanau, Germany) at 500 ± 25 °C until ash was obtained. The ash was bleached and dissolved in nitric acid and then an aqueous solution was prepared for the readings in a Varian spectrophotometer (Spectr AA 55B, Agilent, Santa Clara, CA, USA), with a background deuterium correction. The concentrations of the elements were calculated using calibration curves prepared with five different concentrations of K and Na standard solutions (Sodium standard solution, 1000 mg/L Na in 0.5 M nitric acid (from NaNO_3_) Certipur^®^ standard for AAS, Supelco; Potassium standard solution, 1000 mg/L K in 0,5 M nitric acid (from KNO_3_) Certipur^®^ standard for AAS, Supelco^®^).

#### 2.3.4. Analytical Quality Assurance

The quality of analytical methods was assessed using certified reference material for elements and through participation in a proficiency test in the case of proximate composition. The results are presented in Table 2.

### 2.4. Statistical Analysis

Data were analyzed using IBM SPSS Statistics 27 software. The results correspond to the mean ± standard deviation. The comparison between the two variants of each recipe (traditional and modified) was performed using Student’s *t*-test. The comparison of some sensory attributes (descriptive data) among the three modified recipes (containing seaweeds) was carried out by one-way Anova, followed by the Tukey test. Differences with a *p*-value less than 0.05 were considered statistically significant.

## 3. Results and Discussion

This is the first study evaluating the inclusion of edible seaweeds (*Himanthalia elongata* and *Undaria pinnatifida*) in traditional Portuguese recipes. Sea spaghetti (*Himanthalia elongata*) and wakame (*Undaria pinnatifida*) were selected since they are highly valued in Europe due to their excellent nutritional richness, texture properties, and mild taste, with a broad culinary application that includes “natural cuisine” and specialized bakeries and restaurants [1,2]. From a culinary point of view, their versatility, smooth texture, and flavor, in addition to their nutritional benefits, favor introducing seaweeds to the population’s diet by incorporating them in traditional Portuguese recipes [11].

### 3.1. Sea Spaghetti (Himanthalia elongata) and Wakame (Undaria pinnatifida) Composition

Table 3 shows the chemical composition of seaweed, as knowledge of this profile is imperative both to guarantee the provision of its benefits and to determine the risks it may pose to human health; thus, its study is an asset to human nutrition, encourages the development of alternatives for seaweed inclusion in food, and helps to overcome the aversion of some consumers. The main constituents of seaweed can vary depending on the species, place and time of harvest, water temperature, and exposure to waves [38]. The seaweeds differed in macronutrients and ash but were similar in sodium and potassium content (Table 3). *U. pinnatifida* (used in octopus salad and monkfish rice) had more lipid, protein, and ash, and fewer carbohydrates than *H. elongata* (used in stewed cuttlefish with white beans and clams).

### 3.2. Chemical Composition of the Recipes

Table 4 shows no significant difference in moisture, ash, lipid, protein, sodium, and potassium regarding each traditional and modified recipe. Despite removing salt from AT and FC in the modified recipes, the addition of edible seaweeds did not contribute to sodium reduction since wakame and sea spaghetti have high amounts of sodium [24,39], as confirmed by our study (Table 3).

From a nutritional point of view, sea spaghetti and wakame stand out mainly for their high calcium (about 950 mg/100 g and 980 mg/100 g, respectively) and protein (about 6 g/100 g and 17 g/100 g, respectively) content [24,39], which makes them beneficial alternatives to animal-source food products. Despite the slight reduction in the animal-source ingredients in the modified recipes (Table 1), the protein content did not change (Table 4), showing the importance of the use of edible seaweeds. Sea spaghetti and wakame are also high in dietary fiber (about 49% and 40%, respectively), potentially contributing to consumers’ health [39]. However, more studies are necessary to evaluate the recipes’ fiber content and their effects on human health.

Regarding the energy value, significant differences between the traditional and modified recipes were not found. The reported values were 111 ± 1.3 and 111 ± 1.3 kcal/100 g, for SP and SPW; 120 ± 2.4 and 118 ± 0.6 kcal/100 g, for AT and ATW; and 118 ± 0.8 and 116 ± 3.9 kcal/100g, for FC and FCE, showing that the presence of seaweed did not change the energy values of traditional Portuguese recipes. It was also observed that wakame presented lower potassium content than sea spaghetti (Table 3). However, the recipes prepared with wakame had higher potassium content than those prepared with sea spaghetti (Table 5). This occurred due to the high proportion of seaweed used in the recipes that used wakame (Table 1).

Table 6 presents the nutritional contribution of sodium in the recipes; no differences were found between the traditional and modified recipes. Despite the high amounts of sodium in seaweeds, these recipes used rehydration (1 seaweed: 10 water, corresponding to 5 to 7.5 times the initial weight of sea spaghetti and wakame, respectively, at the end of the rehydration process), which diluted the sodium content. It is also important to highlight that the proportion of edible seaweeds used in the modified recipes was higher than the proportion of salt in the traditional recipes (except for the octopus salad, in which salt was not used) (Table 1). Different from salt (NaCl), edible seaweeds present antioxidant, anticancer, anticoagulant, anti-inflammatory, antidiabetes, and antimicrobial properties, mainly attributed to polysaccharides, carotenoids, tocopherols, phycocyanins, vitamins, fatty acids, and sterols, as well as their amino acids, peptides, and proteins [24,39,41,42]. In this sense, using edible seaweeds in traditional Portuguese recipes might contribute to consumers’ health with no increase in sodium.

### 3.3. Characteristics of the Recipes

**Figure 1:**
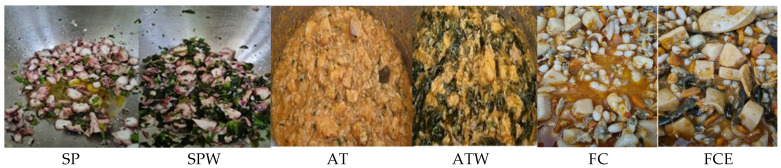
Traditional and modified versions of the recipes. (SP: octopus salad; SPW: octopus salad with wakame; AT: monkfish rice; ATW: monkfish rice with wakame; FC: stewed cuttlefish with white beans; FCE: stewed cuttlefish with white beans and sea spaghetti).

Figure 1 shows the traditional and modified versions of the recipes. Qualitatively, the perception of seaweed in the recipe was essentially visual, making it more challenging to perceive sea spaghetti in SPW and FCE due to the color of the traditional recipe. Due to the seaweed color, the modified versions showed darker colors than the traditional ones. In the octopus salad and monkfish rice, the seaweed used was wakame (*Undaria pinnatifida*). Wakame occupies second place in the world ranking of the seaweed most used in direct feeding. From a culinary point of view, it has a smooth texture and fine flavor, making it one of the most suitable species for introducing seaweeds to the diet [1,11,30,41,44]. Sea spaghetti was used in the stewed cuttlefish with white beans since its flavor is reminiscent of cuttlefish, with a fleshy texture and smooth taste [11,30].

### 3.4. Quantitative Descriptive Sensory Analysis

**Figure 2:**
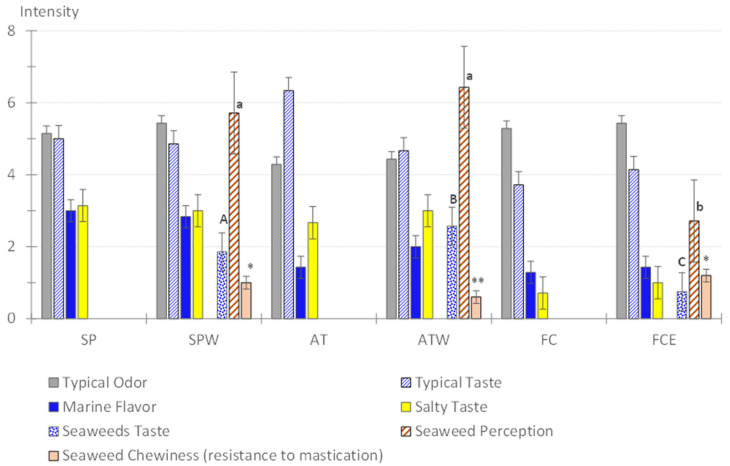
Sensory properties of the traditional and modified recipes. The results are the mean values (n = 8 panelists). SP: octopus salad; SPW: octopus salad with wakame; AT: monkfish rice; ATW: monkfish rice with wakame; FC: stewed cuttlefish with white beans; FCE: stewed cuttlefish with white beans and sea spaghetti). Intensity scale: 0—absent; 1—absent to slight; 2—slight; 3—slight to moderate; 4—moderate; 5—moderate to strong; 6—strong; 7—strong to extreme; 8—extreme. Student’s *t*-test was applied to compare both variants (traditional and modified) of each recipe. Differences were not statistically significant. (*p* > 0.05). One-way Anova, followed by the Tukey test, was applied to compare the three modified recipes (contain seaweeds) SPW, ATW and FCE: capital letters correspond to significant differences in seaweed taste; lowercase letters correspond to significant differences in seaweed perception; * and ** correspond to significant differences in seaweed chewiness (*p* < 0.05).

There were no significant differences in the typical odor and taste of each recipe with the inclusion of seaweeds (Figure 2). The marine flavor was not significantly intensified by including seaweed in the recipes. Even in the case of SPW (containing raw rehydrated wakame), the marine flavor was classified as slight to moderate, as in the SP variant.

Nonetheless, the descriptor seaweed taste was differently perceived among the modified recipes, particularly in the two containing wakame, although with low intensity: slight (mean score = 2) in the case of SPW (with raw rehydrated wakame) and slight to moderate (mean score = 3) in the case of ATW. This indicates that cooking wakame enhanced the seaweed taste. In the case of the FCE recipe, containing sea spaghetti, seaweed taste was perceived only as absent to slight (mean score = 0.8).

The lowest seaweed perception reported for the FCE recipe was mainly due to the color and soft texture resembling green beans. The salty taste was not significantly different by including seaweed in any of the recipes, probably due to the similar sodium content comparing the traditional and modified recipes (Table 3). This attribute was classified as slight to moderate (mean score = 3), except in the case of FC and FCE, which were classified as absent to slight (mean score = 1). There was no noticeable bitter taste in any of the recipes, nor negative mouthfeels such as astringency.

Regarding the texture of the seaweed, both species presented interesting textural properties, namely lack of adhesiveness (the seaweeds did not “stick” to the teeth or palate) and low chewiness (resistance to mastication and swallowing), even with the raw wakame in the SPW recipe (average value of 1—seaweed extremely easy to chew and swallow).

Based on the above results, the modified recipes did not require final adjustments to be subjected to an acceptance test with the nontrained panelists.

### 3.5. Acceptance Test

**Figure 3:**
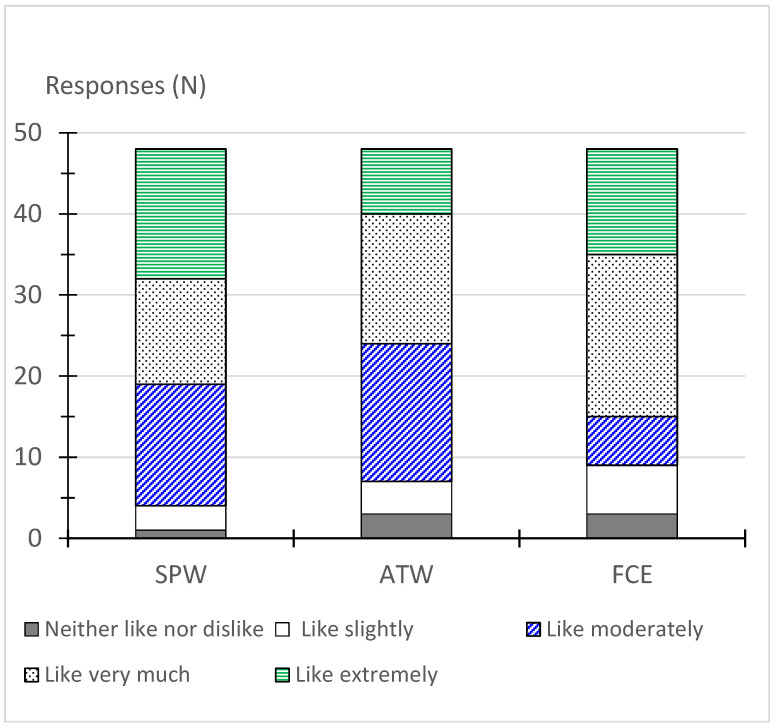
Overall liking (acceptance) of the modified recipes. The results are the number of responses (n = 48 nontrained panelists). SPW: octopus salad with wakame; ATW: monk-fish rice with wakame; FCE: stewed cuttlefish with white beans and sea spaghetti. Hedonic scale: 1—dislike extremely; 2—dislike very much; 3—dislike moderately; 4—dislike slightly; 5—neither like nor dislike; 6—like slightly; 7—like moderately; 8—like very much; 9—like extremely.

Figure 3 shows the overall liking of the modified recipes. No responses were obtained for negative categories (below *neither like nor dislike*) for any recipe. All the modified recipes showed very good acceptance, relatively above 70% [34]: overall, 81, 85, and 92% of the responses ranged from *like moderately* to *like extremely*, in the case of FCE, ATW, and SPW, respectively. However, the SPW and FCE recipes were the most appreciated since 60 and 69% of the responses were for the categories of *like very much* and *like extremely*, respectively.

Negative odors, flavors/taste, texture, or mouthfeel were not perceived in any modified recipe. Regarding what the panelists most liked in each modified recipe, the main findings were the following:

SPW—Overall, 79.2% of the respondents highlighted the marine-like flavor, and somewhat bivalve flavor, and 45.8% of the respondents indicated the perception of the seaweed’s texture in contrast with the tenderness of cooked octopus. The panelists stated that raw wakame (rehydrated) added an interesting crunchy texture to the recipe.

ATW—Overall, 66.7% of the respondents emphasized the attractive and harmonious appearance of the recipe and marine-like flavor, and 33.3% also referred to the seaweed’s texture. Even when cooked, wakame still presented a slightly crunchy texture, which was appreciated by the panelists.

FCE—Overall, 72.9% of the respondents pointed to the marine-like flavor and seaweed taste, 52.1% referred to the soft seaweed’s texture (sea spaghetti) like green beans, and 45.8% of the respondents also highlighted the attractive and harmonious appearance of the recipe.

### 3.6. Strengths and Limitations

This study has both strengths and limitations. Despite its strengths, the potential nutritional benefits and panelists’ positive acceptance can be enhanced, as some of the limitations observed cannot be overlooked. In the case of octopus salad with wakame, salt reduction could not be considered, as octopus already has a considerable amount of sodium in its composition [31]. Also, due to the limitation in laboratory analysis, we could not analyze the recipes’ fiber and bioactive compound content. More studies are necessary to compare fiber and bioactive compounds from traditional and modified recipes and their effects on consumers’ health.

## 4. Future Perspectives

With a view to future work, it is important to define the microbiological rate of organisms allowed in human food, as well as to determine the extent of seaweed’s health risks regarding their metal and iodine content. So, it is important to know seaweed’s life cycle and bioavailability. Other studies show that processing vegetables for consumption exposes the phytochemicals present to harmful factors that can lead to changes in concentrations and health-related quality. Because seaweed is consumed after some kind of processing, it is important to investigate the effect of such treatments on the bioactive compounds present in seaweed [45,46].

In future studies, it is important to carry out more chemical analyses to corroborate the nutritional benefits, specifically regarding selenium, fiber, and the bioactivity of seaweeds after culinary processes, as well as the rehydration water of dried seaweed.

Knowing the best culinary processes to use is also crucial since access to other components present in seaweeds is only possible by breaking down the cell wall [47]. No less important is understanding the effects of cooking processes on the chemical components present in seaweeds.

## 5. Conclusions

This is the first study on the sensory and nutritional aspects of traditional Portuguese recipes using edible seaweeds, and the findings showed that edible seaweeds were well accepted by a nontrained sensory panel and did not change nutritional aspects of macronutrients, ash, and sodium. However, wakame contributed to an increase in the potassium content in octopus salad and monkfish rice.

The utilization of functional ingredients may be a sustainable and healthy alternative in order to innovate traditional recipes, reducing the use of salt. In particular, the use of raw or even slightly heat-treated wakame can enhance the bioactivity of the ingredients used in the traditional octopus salad. In short, the results of sensory analysis highlighted the potential for seaweed inclusion in Portuguese traditional recipes without compromising its identity while promoting the use of more sustainable ingredients with nutritional benefits, in particular, with the reduction in salt. Considering the sodium, potassium, protein, and carbohydrate content, in future work, the partial substitution of fish/mollusks by seaweeds should be evaluated to improve the sustainability of these recipes. It is necessary to adjust the recipe formulation, particularly the amount of seaweed to be added, considering the characteristics of each seaweed species and the culinary process. More chemical analyses should be performed to corroborate seaweed’s nutritional benefits, specifically its iodine, selenium, and fiber content.

## Figures and Tables

**Table 1:** Ingredients used in each recipe (traditional and modified).

Recipe	Ingredients	% of Each Ingredient	
		Traditional Recipe	Modified Recipe
*Octopus Salad*	Cooked octopus (boiled, without salt; white onion, bay leaves and garlic)	**SP** 63.42	**SPW** 55.25
	Onion (purple)	10.68	9.21
	Green pepper	8.55	7.37
	Freshly ground pepper	0.11	0.09
	Fresh coriander	0.53	0.46
	Olive oil	10.68	9.21
	White wine vinegar	5.34	4.60
	Salt	-	-
	Wakame (*U. pinnatifida*)- Rehydrated	-	13.81
Monkfish Rice with Prawns	Monkfish (cubes)	**AT** 27.76	**ATW** 26.54
	Prawn tails	9.25	8.85
	Onion	6.94	6.63
	Garlic	0.46	0.44
	Freshly ground pepper	0.05	0.04
	White whine	4.63	4.42
	Olive oil	2.31	2.21
	Carolino rice	11.57	11.06
	Tomato	2.31	2.21
	Seafood broth (olive oil prawn heads, monkfish spines, salt, onion, water)	34.71	33.17
	Salt	0.18	-
	Wakame (*U. pinnatifida*)-rehydrated	-	4.42
Stewed cuttlefish with white beans and clams	Cuttlefish (cubes)	**FC** 20.70	**FCE** 20.04
	Cooked white beans (boiled, without salt)	27.61	26.72
	Clam kernel	6.90	6.68
	Onion	2.76	2.67
	Garlic	0.17	0.17
	Freshly ground pepper	0.07	0.07
	White bean cooking water	20.70	20.04
	White wine	3.45	3.34
	Parsley	0.17	0.17
	Carrot	5.18	5.01
	Olive oil	1.73	1.67
	Tomato	6.90	6.68
	Bay leaves	0.03	0.03
	Cumin powder	0.03	0.03
	Tomato paste	3.45	3.34
	Salt	0.14	-
	Sea spaghetti (*H. elongata*) rehydrated—pieces	-	3.34

SP: *octopus salad*; SPW: *octopus salad with wakame*; AT: *monkfish rice*; ATW: *monkfish rice with wakame*; FC: *stewed cuttlefish with white beans*; FCE: *stewed cuttlefish with white beans and sea spaghetti*.

**Table 2:** Quality assurance of the proximates and macroelement analyses (n = 4).

Component	Method	LOQ	Proficiency Test or CRM	Certified Value	Present Work
Proximates (g/100 g)					
Moisture	Drying	0.07	FAPAS 25234 ^a^	65.6 ± 0.5 ^b^	65.7 ± 0.3 ^c^
Ash	Incineration	0.18	FAPAS 25234 ^a^	3.82 ± 0.12 ^b^	3.81 ± 0.19 ^c^
Fat	Soxhlet	0.3	FAPAS 25234 ^a^	13.6 ± 0.5 ^b^	13.5 ± 0.8 ^c^
Nitrogen	Combustion	0.002	FAPAS 25234 ^a^	2.59 ± 0.05 ^b^	2.60 ± 0.02 ^d^
Macroelements (mg/kg)					
Potassium	FAAS	0.07	SQID-1 ^e^	4660 ± 1420 ^c^	5181 ± 202 ^d^
Sodium	FAAS	0.30	SQID-1 ^e^	14,600 ± 1600 ^c^	13,565 ± 189 ^d^

^a^ Nutritional Components in Fish Paste—FAPAS Food Chemistry Proficiency Test Report 25234; ^b^ Standard deviation for proficiency, σp; ^c^ expanded uncertainty (k = 2); ^d^ standard deviation; ^e^ cuttlefish certified reference material for trace metals, arsenobetaine, and methylmercury (date of expiry: July 2028) from NRC (National Research Council Canada); FAAS—flame atomic absorption spectrometry.

**Table 3:** Chemical composition of the edible dehydrated seaweeds.

Seaweeds	Moisture g/100 g	Lipid g/100 g	Ash g/100 g	Protein g/100 g	Carbohydrate g/100 g *	Potassium mg/100 g	Sodium mg/100 g
*U. pinnatifida* (wakame)	11.5 ± 0.1 ^a^	2.3 ± 0.0 ^a^	35.07 ± 0.39 ^a^	13.9 ± 0.4 ^a^	37.2 ± 0.7 ^a^	5589.3 ± 1376.0 ^a^	6383.2 ± 1119.9 ^a^
*H. elongata* (sea spaghetti)	12.1 ± 0.0 ^b^	1.6 ± 0.1 ^b^	30.84 ± 0.06 ^b^	10.7 ± 0.00 ^b^	44.7 ± 0.2 ^b^	7089.6 ± 929.7 ^a^	5530.6 ± 510.6 ^a^

* Calculated by difference [36]: % Total carbohydrates=100−(% moisture+% fat+% ashes+% protein). In each column, different superscript letters correspond to significant differences (Student’s *t*-test, *p* < 0.05).

**Table 4:** Chemical composition of traditional and modified recipes (wet weight).

Recipes	Moisture g/100 g	Lipid g/100 g	Ash g/100 g	Protein g/100 g	Carbohydrate g/100 g *	Potassium mg/100 g	Sodium mg/100 g
SP	77.6 ± 0.1 ^a^	6.0 ± 0.2 ^a^	2.09 ± 0.02 ^a^	13.7 ± 0.04 ^a^	0.7 ± 0.2 ^a^	66.9 ± 5.3 ^a^	240.7 ± 24.4 ^a^
SPW	77.7 ± 0.3 ^a^	6.0 ± 0.3 ^a^	2.58 ± 0.03 ^a^	12.2 ± 0.2 ^a^	1.5 ± 0.4 ^b^	157.0 ± 26.5 ^b^	252.4 ± 32.7 ^a^
AT	73.4 ± 0.5 ^a^	3.5 ± 0.1 ^a^	0.97 ± 0.01 ^a^	9.0 ± 0.2 ^a^	13.1 ± 0.5 ^a^	176.1 ± 4.9 ^a^	120.6 ± 6.8 ^a^
ATW	74.7 ± 0.3 ^a^	4.2 ± 0.2 ^a^	1.11 ± 0.01 ^a^	8.7 ± 0.1 ^a^	11.3 ± 0.3 ^b^	219.7 ± 21.4 ^b^	112.5 ± 11.4 ^a^
FC	73.5 ± 0.1 ^a^	3.5 ± 0.2 ^a^	1.38 ± 0.02 ^a^	11.1 ± 0.7 ^a^	10.4 ± 0.9 ^a^	227.3 ± 11.2 ^a^	181.6 ± 20.2 ^a^
FCE	74.4 ± 0.1 ^a^	4.1 ± 0.8 ^a^	1.58 ± 0.01 ^a^	9.0 ± 0.6 ^a^	10.9 ± 0.4 ^a^	255.8 ± 34.8 ^a^	173.4 ± 18.7 ^a^

* Calculated by difference [36]. In each column, for each recipe (traditional and modified), different superscript letters correspond to significant differences (Student’s *t*-test, *p* < 0.05). SP: *octopus salad*; SPW: *octopus salad with wakame*; AT: *monkfish rice*; ATW: *monkfish rice with wakame*; FC: *stewed cuttlefish with white beans*; FCE: *stewed cuttlefish with white beans and sea spaghetti.*

**Table 5:** Potassium nutritional contribution (NC, %) of the recipes, considering the usual portion of a complete dish.

Population	AI	NC (%) of Recipes ^2^					
Groups	(mg/Day) ^1^	SP	SPW	AT	ATW	FC	FCE
7–10 years	1800	7.4 ± 0.6 ^a^	17.4 ± 2.9 ^b^	29.3 ± 0.8 ^a^	36.6 ± 3.6 ^b^	37.9 ± 1.9 ^a^	42.6 ± 5.8 ^a^
11–14 years	2700	5.0 ± 0.4 ^a^	11.6 ± 2.0 ^b^	19.6 ± 0.5 ^a^	24.4 ± 2.4 ^b^	25.3 ± 1.2 ^a^	28.4 ± 3.9 ^a^
15–17 years	3500	3.8 ± 0.3 ^a^	9.0 ± 1.5 ^b^	15.1 ± 0.4 ^a^	18.8 ± 1.8 ^b^	19.5 ± 1.0 ^a^	21.9 ± 3.0 ^a^
≥18 years ^3^	3500	3.8 ± 0.3 ^a^	9.0 ± 1.5 ^b^	15.1 ± 0.4 ^a^	18.8 ± 1.8 ^b^	19.5 ± 1.0 ^a^	21.9 ± 3.0 ^a^
Lactating women	4000	3.3 ± 0.3 ^a^	7.8 ± 1.3 ^b^	13.2 ± 0.4 ^a^	16.5 ± 1.6 ^b^	17.0 ± 0.8 ^a^	19.2 ± 2.6 ^a^

^1^ EFSA [40]. ^2^ Portions of 200 g for SP/SPW and 300 g for the other recipes. ^3^ Including pregnant women. In each row, for each recipe (traditional and modified), different superscript letters correspond to significant differences (Student’s *t*-test, *p* < 0.05).

**Table 6:** Sodium nutritional contribution (NC, %) of the recipes, considering the usual portion of a complete dish.

Population	AI	NC (%) of Recipes ^2^					
Groups	(g/Day) ^1^	SP	SPW	AT	ATW	FC	FCE
7–10 years	1.7	25.7 ± 7.1 ^a^	29.7 ± 3.8 ^a^	21.3 ± 1.2 ^a^	18.3 ± 4.4 ^a^	32.1 ± 3.6 ^a^	30.6 ± 3.3 ^a^
11–17 years	2.0	21.8 ± 6.0 ^a^	25.2 ± 3.3 ^a^	18.1 ± 1.0 ^a^	15.5 ± 3.7 ^a^	27.2 ± 3.0 ^a^	26.0 ± 2.8 ^a^
≥18 years ^3^	2.0	21.8 ± 6.0 ^a^	25.2 ± 3.3 ^a^	18.1 ± 1.0 ^a^	15.5 ± 3.7 ^a^	27.2 ± 3.0 ^a^	26.0 ± 2.8 ^a^

^1^ EFSA [43]. ^2^ Portions of 200 g for SP/SPW and 300 g for the other recipes. ^3^ Including pregnant and lactating women. In each row, for each recipe (traditional and modified), different superscript letters correspond to significant differences (Student’s *t*-test, *p* < 0.05).

## Data Availability

The original contributions presented in this study are included in the article. Further inquiries can be directed to the corresponding author.
